# Assessment of the psychometrics of the Students' Attitudes towards Addressing Sexual Health Extended (SA-SH-Ext) questionnaire for social educator students

**DOI:** 10.1016/j.esxm.2022.100507

**Published:** 2022-04-03

**Authors:** Gerd Hilde Lunde, Laila Blaalid, Helle Gerbild, Kristina Areskoug Josefsson

**Affiliations:** 1 Department for Behavioral Sciences, Oslo Metropolitan University, Oslo, Norway; 2 Health Science Research Centre, UCL University College, Odense, Denmark; 3 VID Specialized University, Faculty of Health Studies, Sandnes, Norway

**Keywords:** Psychometrics, Reliability, Sexual Health, Social Educator, Validity

## Abstract

**Background:**

Social educators have an important role in promoting and rehabilitating sexual health as they provide information, discuss, and support sexual health in daily life for persons living with impairments, intellectual disabilities, and complex needs.

**Aim:**

The study aimed to assess the psychometrics of the Students´ Attitudes towards addressing Sexual Health Extended (SA-SH-Ext) questionnaire for social educator students.

**Methods:**

A sample of 213 Norwegian social educator students was used to test internal consistency reliability and construct validity with explorative factor analysis.

**Outcomes:**

Internal consistency reliability showed a Cronbach's alpha of 0.906 and construct validity measured with explorative factor analysis showed good results with the Kaiser-Meyer-Olkin measure of sampling adequacy (KMO) being high (0.929) and Bartlett´s test of sphericity being significant (*P* = .000).

**Results:**

The SA-SH-Ext is reliable and valid for social educator students, however the psychometric assessment revealed that the domains of the SA-SH-Ext should be revised compared to the original SA-SH questionnaire.

**Clinical implications:**

Measuring the effectiveness of sexual health education interventions is important and to have a valid and reliable questionnaire to assess future professionals’ attitudes towards addressing sexual health increases the ability to target specific client needs or knowledge gaps, such as addressing sexual health for persons with intellectual disabilities.

**Strengths:**

and limitations: In comparison with previous studies of the original SA-SH, SA-SH-Ext has high reliability and validity. The current study was performed with classical test theory. Performing Rasch analysis may detect other psychometric issues, by improving precision and thereby providing a deeper understanding of both how to optimise a questionnaire and understand the results of a used questionnaire. Despite the response rate of 34%, the results are seen as valid considering the low correlation between response rate and validity and that the sample size was sufficient for the chosen psychometric tests.

**Conclusion:**

The SA-SH-Ext is a valuable questionnaire for assessing the level of perceived preparedness among social educator students in addressing sexual health, a field often neglected in health and care.

**Lunde GH, Blaalid L, Gerbild H, et al. Assessment of the psychometrics of the Students' Attitudes towards Addressing Sexual Health Extended (SA-SH-Ext) questionnaire for social educator students. Sex Med 2022;10:100507.**

## What do we already know about this topic?


 

• Clients´ sexual health needs are often insufficiently met, which affects quality of life and well-being.
• Social educators have an important role in promoting and rehabilitating sexual health and there is a need to be able to assess social educator students´ readiness and attitudes towards addressing sexual health in their future profession to ensure effectiveness of educational interventions and sexual health support for future clients.

## What does this study add?


 

• The Students´ Attitudes towards addressing Sexual Health Extended (SA-SH-Ext) questionnaire is useful, valid, and reliable to measure social educator students´ readiness to address sexual health issues with future clients.

## INTRODUCTION

Students in professional training and higher education are supposed to be prepared for meeting the challenges of their future profession. However, concerning sensitive topics like sexual health, their personal beliefs, staff narratives and the attitudes of students may have a greater impact on their future profession than in other areas,^1^ for example concerning sexuality for non-heterosexual persons living with intellectual disabilities.^2^ Research shows that professionals in health and welfare are reluctant to address sexual health issues, and clients´ needs are insufficiently met.^3^ There are several reasons why health care professionals do not address sexual health, such as lack of competence, feelings of taboo, fear of over-stepping private boundaries, insecurity, embarrassment, insufficient education, thinking the topic is unimportant to the clients, lack of time and lack of clinical guidelines in this field.^3^, ^4^, ^5^, ^6^, ^7^, ^8^, ^9^, ^10^, ^11^, ^12^ However, the Norwegian ratification of the Convention of the Rights of Persons with Disabilities^13^ means that Norway is part of an international commitment to grant everyone with disabilities the same rights as the rest of the population^14^ including rights related to sexual health. The Norwegian Strategy for Sexual Health^15^ focuses on the importance of openness, positive attitudes, and respect for diversity to promote sexual health. The strategy is intended to protect the individual´s sexual rights and the sexual needs of clients.^15^ Cultural context affects beliefs and attitudes regarding sexuality and sexual health.^16^, ^17^, ^18^ Staff has an important role in assisting clients´ meet their needs related to sexual issues,^1^^,^^8^ and social educators should facilitate understanding for other health care professionals on how to assist in meeting clients´ health needs through the lifespan.^5^^,^^19^^,^^20^ Despite this, social educators can feel uncomfortable when teaching clients with intellectual disabilities about the body, identity, and sexual health, and may feel they lack support from management and colleagues when teaching in this area.^21^

Social educators work with a life cycle perspective with persons of all ages in a broad range of activities and organizations in Norway.^5^^,^^20^^,^^22^^,^^23^ The professional scope of social educators shows their important role in promoting and rehabilitating sexual health for persons living with disabilities,^5^ a role which includes the obligation to provide information, discuss and support sexual health in daily life for persons living with disabilities and with complex needs.^21^ Many of the social educators´ clients are vulnerable due to their disabilities^20^ and at risk of missing out on ordinary sexual health promotive interventions in society, despite sexual health being important for quality of life.^2^ Persons living with intellectual disabilities are a large group of clients for social educators,^5^^,^^20^^,^^22^ and they have a higher risk of sexual abuse.^24^ Persons living with intellectual disabilities also risk being sexual abusers themselves,^8^^,^^25^ due to lack of comprehension of social boundaries^7^ and because persons with intellectual disabilities are rarely afforded comprehensive rights related to their sexuality.^26^ Several areas are important to address to support persons with intellectual disabilities, such as for example sexual relationships, contraception, pregnancy, taking responsibility for sexual behaviour and sexually transmitted diseases.^1^ In addition, service users with intellectual disabilities in care settings experience significant restrictions on pursuing intimate relationships,^1^ further indicating the importance of ensuring professional competence in this field.^18^ Empathy, trust, self-efficacy, and ethical reflection are core concepts for social educators.^21^ Those qualifications are especially important concerning sensitive topics like sexual health and intellectual disability since professionals are ambivalent towards respecting the sexual rights of persons with intellectual disability.^6^ There is a need for education of professionals to provide qualified support for persons with intellectual disabilities concerning sexual health.^1^

There are many reasons why professionals do not address sexual health, and these also apply to students in health professional educations.^27^, ^28^, ^29^, ^30^, ^31^ recent study has shown that only 11% of health care students had knowledge of sexual health for persons with disabilities, and most of the students considered the topic to be overlooked in society.^32^ Teaching students how to be prepared for their future profession is not only an issue of providing professional and theoretical knowledge; the students also need to learn how to implement the knowledge in practice to be able to perform the tasks included in their professional role. The teachers must understand and address sensitive topics at the level of understanding that the students must enhance the skills they will need in practice. This stresses the importance of having reliable and valid questionnaires to assess students´ attitudes and perceived competence concerning sexual health, so the assessments can lead to further development of social educator programmes and thereby improve future care.

There have been studies measuring health care professional students´ attitudes towards addressing sexual health using the Students´ Attitudes towards addressing Sexual Health (SA-SH) questionnaire,^28^^,^^31^^,^^33^, ^34^, ^35^, ^36^, ^37^, ^38^, ^39^, ^40^, ^41^ however not in Norway or with social educator students. Using previously validated and utilised questionnaires is resource-saving but may have limited suitability in the context being explored. The SA-SH has been translated into several languages, and an extended Norwegian version (SA-SH-Ext) more suitable for social educators has been developed and tested for content validity.^42^ The adaptations in the SA-SH-Ext consisted of adding 5 items to the original SA-SH, covering the students´ attitudes towards addressing sexual health with clients living with physical or intellectual disabilities and/or diseases, and the students´ self-efficacy in addressing sexual health. However, the SA-SH-Ext needs to be further assessed regarding reliability and validity to ensure its usefulness for research and educational interventions. Therefore, even though the psychometric properties were strong in the original validated questionnaire, psychometric testing is essential, since there is never a certainty that the same psychometric properties will apply in the novel context.^43^ Insufficient psychometric assessment of questionnaires may lead to missing data and decreased response rates due to difficulties in responding to the questionnaire or the risk of drawing questionable conclusions from the collected data.^43^

## AIM

The aim of the study was to assess the psychometrics of the Students´ Attitudes towards addressing Sexual Health Extended (SA-SH-Ext) for social educator students.

## METHODS

The psychometric assessment was performed on a sample of social educator students in Norway. The reporting of this study is guided by the COSMIN group's definitions and taxonomy of measurement properties.^44^^,^^45^

### Sample

All social educator students enrolled at a social educator programme at a Norwegian University were invited to participate (630 students, both part-time and full-time students). The response rate was 34%, with 213 respondents. The respondents´ age range was 19-55 years (median 25 years). The respondents were 164 women, 48 men and 1 respondent defined their gender as other.

### Data Collection Procedure

The data was collected by using an online version of the SA-SH-Ext from December 2019 to January 2020. Information about the study and a link to the online questionnaire was sent to the students´ university mail addresses at the beginning of December. A reminder e-mail was sent 4 weeks after the first invitation to participate in the study.

### Questionnaire

The SA-SH-Ext consists of 27 items, covering the same 4 domains as the original SA-SH: present feelings of comfortableness, future working environment, fear of negative influence on future patient relations, and educational needs. The SA-SH-Ext has 5 additional items compared to the original SA-SH, 4 items in the domain Present feelings of comfortableness and 1 item in the domain educational needs.^38^^,^^42^ All items are measured by using a 5 step Likert scale (disagree, partly disagree, partly agree, agree, strongly agree). The responses ‘strongly agree and/or agree’ are considered positive for positively loaded items, and for negatively loaded items the responses ‘disagree and/or partly disagree’ are considered as showing a negative attitude. The response “partly agree” is not categorized as positive or negative, based on the discrimination of response options in the Rasch-analysis of the SA-SH.^35^ Items 13-18 and 20-22 are reversed for analysis, as these items are phrased negatively compared to all other items.^38^ Descriptive questions related to gender, age, and educational level within the programme are also included.

### Ethics

The study was approved by the department board at the Department of Behavioural Science, Oslo Metropolitan University on 28 November, 2019. All invited students received written information about the study. Before answering the SA-SH-Ext, the students gave their informed consent to participate in the first part of the online questionnaire.

### Analysis

Reliability assessment was performed by measuring internal consistency reliability with Cronbach's alpha, with a Cronbach's alpha of 0.70–0.95 considered as good.^46^

Construct validity was assessed with explorative factor analysis, with principal component as the extraction method and for factor rotations Varimax with Kaiser normalisation was used. Items with high factor loadings defined each dimension. To be a clinically meaningful item in 1 of these factors, each item had to have a loading over 0.50.^47^ Each item was referred to the factor in which it had the highest loading. A scree plot was used to determine the optimal number of factors. Both the Kaiser-Meyer-Olkin measure of sampling adequacy and Bartlett´s test of sphericity were used to ensure the usefulness of factor analysis for the collected data. A value close to 1.0 on the Kaiser-Meyer-Olkin measure of sampling adequacy indicated the proportion of variance of variables that might be caused by underlying factors, and a value below 0.05 on Bartlett´s test of sphericity was used as a level to indicate that factor analysis was suitable.

The limit of statistical significance was set at a = 0.05. The statistical analyses were performed with IBM SPSS version 26 (IBM Corp, Armonk, NY, USA).

## RESULTS

Internal consistency reliability with Cronbach's alpha showed very good results, with a Cronbach's alpha of 0.906. Cronbach´s alpha was between 0.895 and 0.914 even if any of the items were deleted. The internal consistency reliability test shows that all new items should be kept in the SA-SH-Ext

All response options were used for all items; however, items 11, 24 and 27 had most answers at the level “totally agree.”

Construct validity was measured with explorative factor analysis and showed good results. The Kaiser-Meyer-Olkin measure of sampling adequacy (KMO) was high (0.929) and Bartlett´s test of sphericity was significant (*P* = .000). This means that the analysis presents a high explanatory level for variance in the responses.

**Figure 1:**
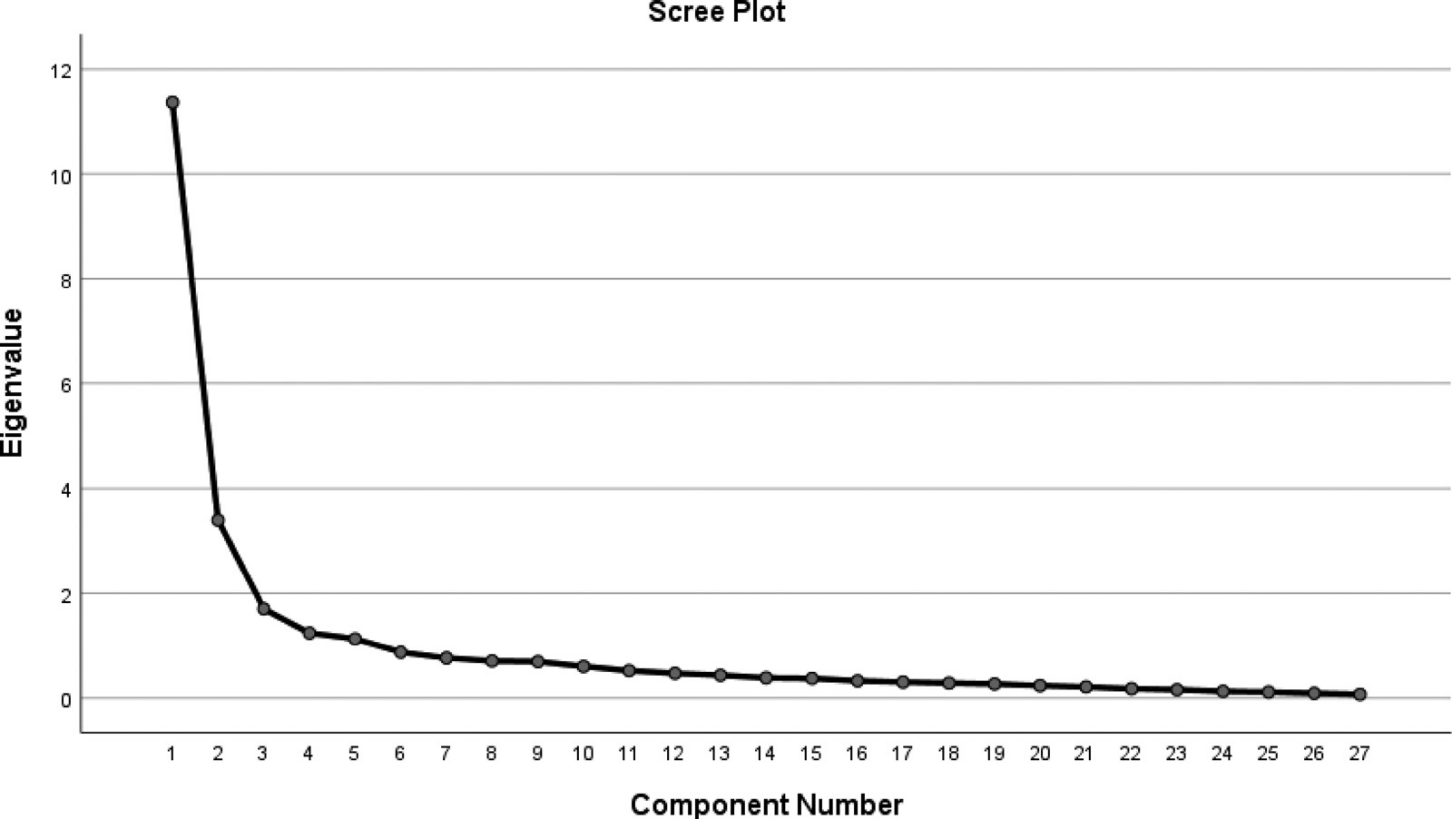
Scree plot of factors.

In Figure 1 the factor analysis (eigen value > 0.5) led to 5 factors, where the first factors (factors 1 and 2) were the most stable (Figure 1).

Table 1 presents the loading of the items in the factors. Items 1-12 were in factor 1, items 13-18 in factor 2, items 20-22 in factor 3, items 23 and 26 in factor 4, items 24 and 27 in factor 5. Items 19 and 25 did not load in any of the factors since the Eigen values were < 0.5. The items in the factors were not identical to the distribution of the items in the 4 domains of the SA-SH-Ext. The factors explain 69.7% of the variances in the factor analysis, which is a reasonable result.

**Table 1:** Rotated component matrix for the 5 factors

Item		Component				
		1	2	3	4	5
1	I feel comfortable about informing future clients about sexual health.	0.852				
2	I feel comfortable about initiating a conversation regarding sexual health with future clients.	0.834				
3	I feel comfortable about discussing sexual health with future clients.	0.877				
4	I feel comfortable about discussing sexual health issues with future clients with physical disability.	0.920				
5	I feel comfortable about discussing sexual health issues with future clients with physical disease.	0.897				
6	I feel comfortable about discussing sexual health issues with future clients with intellectual disability.	0.900				
7	I feel comfortable about discussing sexual health issues with future clients with mental illness.	0.887				
8	I feel comfortable about discussing sexual health issues with future clients, regardless of their sex.	0.877				
9	I feel comfortable about discussing sexual health issues with future clients, regardless of their age.	0.829				
10	I feel comfortable about discussing sexual health issues with future clients, regardless of their cultural background.	0.851				
11	I feel comfortable about discussing sexual health issues with future clients, regardless of their sexual orientation.	0.866				
12	I feel comfortable about discussing specific sexual activities with future clients.	0.797				
13	I am unprepared to talk about sexual health with future clients.		0.599			
14	I believe that I might feel embarrassed if future clients talk about sexual issues.		0.684			
15	I believe that future clients might feel embarrassed if I bring up sexual issues.		0.610			
16	I am afraid that future clients might feel uneasy if I talk about sexual issues.		0.696			
17	I am afraid that conversations regarding sexual health might create a distance between me and the clients.		0.719			
18	I believe that I will have too much to do in my future profession to have time to handle sexual issues.		0.531			
19	I will take time to deal with clients’ sexual issues in my future profession.					
20	I am afraid that my future colleagues would feel uneasy if I brought up sexual issues with clients.			0.693		
21	I am afraid that my future colleagues would feel uncomfortable dealing with questions regarding clients’ sexual health.			0.865		
22	I believe that my future colleagues will be reluctant to talk about sexual issues.			0.813		
23	In my education I have been educated about sexual health.					0.800
24	I think that I, as a student, need to get basic knowledge about sexual health in my education.				0.836	
25	I have sufficient competence to talk about sexual health with my future clients.					
26	I believe in my own ability to promote sexual health in my future profession.					0.520
27	I think that I need to be trained in my education to talk about sexual health.				0.776	

Extraction method: Principal Component Analysis.

Rotation method: Varimax with Kaiser Normalisation.

A Rotation converged in 6 iterations.

## DISCUSSION

The reliability of the SA-SH-Ext is very good, and the added items fit well in the extended questionnaire. The results from this study together with the previous content validity test^42^ show that the SA-SH-Ext has good psychometric quality as defined by COSMIN guidelines.^44^^,^^45^ The SA-SH-Ext is simple and quick, and thus the questionnaire can give guidance on both the students´ level of readiness and on the need for additional education to provide sufficient competence in the field of sexual health in social educator programmes. The SA-SH-Ext has broader coverage than the original SA-SH, which makes the SA-SH-Ext suitable for the health and welfare professions, especially considering the added focus on disability and/or disease and sexual health. The ability to evaluate social educator students´ level of competence may give guidance on how to develop education to ensure that the Convention of the Rights of Persons with Disabilities includes sexual health in practice. Professionals, such as social educators, who are skilled in sexual health, can assist in enabling everyone´s attainment of the sexual citizenship and sexual rights that they are legally and morally entitled to.^48^ In social educator programmes in Norway, education on sexual health differs, indicating that among students there is a risk of different levels of readiness to address sexual health in their future profession. If the students are insufficiently prepared, previously described hindrances^8^^,^^10^^,^^21^ together with staff narratives^18^ may further prevent clients´ needs within sexual health being met.

Prior to this study, research had shown that the face validity and content validity of the SA-SH-Ext were high,^42^ and the usefulness of the SA-SH in other countries and contexts also implies the potential value of the questionnaire.^28^^,^^31^^,^^34^^,^^36^^,^^39^^,^^40^ However, an additional psychometric assessment was essential to ensure the value of the SA-SH-Ext, since face validity is questioned as a psychometrically useful test.^49^^,^^50^ The psychometric tests showed good internal consistency of the SA-SH-Ext, thus indicating that the novel items should be kept. The level of Cronbach´s alpha was higher both for the SA-SH-Ext than for the original SA-SH,^38^^,^^40^ and the Danish version of SA-SH,^37^^,^^51^ indicating better reliability for the SA-SH-Ext than for the original SA-SH.

The factor analysis reveals that the domains for the SA-SH-Ext are not completely in line with the factor analysis of the original SA-SH, especially concerning factors 4 and 5.^38^ The high value of sampling adequacy indicates the usefulness of the factor analysis of the SA-SH-Ext. By following the factor analysis of SA-SH-Ext and the experience of using the SA-SH and the SA-SH-EXT in various contexts,^31^^,^^33^^,^^36^^,^^39^^,^^42^ the suggestion is that the domains for SA-SH-Ext should be revised. A proposed new set of domains are the following: (domain 1)” being comfortable,” (domain 2) “future client relations,” (domain 3) “future working relations” and (domain 4) “education & competence. Domain 1 ”being comfortable” includes items 1-12, domain 2 “future client relations” includes items 13-19, domain 3 “future working relations” includes items 20-22 and domain 4 “education & competence includes items 23-27. In this allocation of items, items from factors 4 and 5 are brought together into 1 domain. Item 19 “I will take time to deal with clients’ sexual issues in my future profession” and item 25 “I have sufficient competence to talk about sexual health with my future clients.” did not load to any of the factors; therefore, those items have been added to the domains where they fit best according to the authors' experiences from previous work with the SA-SH. Those 2 items did not load to the 3 major factors in the original SA-SH either,^38^ but were considered to be important to keep in the SA-SH based on qualitative studies and experience from practice.^38^ Items 19 and 25 were also kept since they are in line with previous research describing time and competence as reasons for not discussing sexual health with clients.^3^^,^^9^^,^^10^^,^^12^ For future studies and practical use in other contexts, it is recommended to perform a factor analysis to ensure the stability of the factors. Additional psychometric testing, including factor analysis, when using SA-SH-Ext in novel contexts is also recommended due to the cultural differences and sensitivity regarding sexual health.

Items 11, 24 and 27 had most answers on the level “totally agree.” The high response to item 11 “I feel comfortable about discussing sexual health issues with future clients, regardless of their sexual orientation” is positive, since a welcoming, open attitude regardless of sexual orientation improves communication with non-heterosexual persons.^52^ However, item 11 may not get as high scores in another cultural context and further research is needed to see how this item is responded to in other contexts. Persons identifying as lesbian, gay, bisexual, transgender, and queer (LGBTQ) and living with intellectual disability can experience a double stigma regarding sexuality, and to feel fully supported, they desired holistic service provision sensitive to their sexuality and intellectual disability needs.^2^ In future studies, it would also be of interest to research how professionals consider working with sexual health if the users have combinations of the characteristics mentioned in items 4-11, since this may affect their attitudes and is not covered by the SA-SH-Ext. Items 24 and 27 concern the needs for education about sexual health and needs to be trained to communicate about sexual health, which indicates the importance of including those topics in social educators´ basic education and training. It is also possible that items 24 and 27 are highly rated since social educators are expected to have excellent skills in communication and establishing relations.^19^^,^^53^

In comparison with previous studies of the original SA-SH,^38^^,^^40^^,^^41^ and the Danish version of the SA-SH,^37^^,^^51^ the extended version has high reliability and validity. Measuring the effectiveness of sexual health education interventions is important^18^ and valid and reliable measurements to assess students´ readiness to address sexual health can provide teachers in higher education with a questionnaire to ensure the sustainable value of interprofessional sexual health educational interventions.^33^ The SA-SH-Ext could be a useful questionnaire to evaluate sexual health educational interventions at social educator programmes. To have a valuable questionnaire to assess future professionals’ attitudes towards addressing sexual health increases the ability to target specific client needs or knowledge gaps, such as addressing sexual health for persons with intellectual disabilities. The original SA-SH has been used and psychometrically tested for a variety of forms of professional education, for example for social worker students, occupational therapy students, nursing students, physiotherapy students, prosthetics, and orthotics students. Therefore, it is possible to assume that SA-SH-Ext can be useful for other health and welfare educational programmes as well.

The time for the collection of data may have affected the response rate since it coincided with the Christmas holiday season in Norway. At the same time period, there were students on practice placement, which may also have affected the response rate since the students conducting practice studies do not use their online learning platform or their student e-mail as much as when they have ordinary courses on campus. There is also a risk that some students have quit the programme, but still being on the course participation list. However, response rates are declining for online questionnaires and when the questionnaire addresses a sensitive topic, like sexual health, it is expected that the response rate can be affected. Despite the response rate of 34%, it is still likely that the results are valid considering the low correlation between response rate and validity^54^ and that the sample size was sufficient for the chosen psychometric tests.

The current study was performed with classical test theory. Performing Rasch analysis may detect other psychometric issues affecting a questionnaire apart from those found in classical test theory, by improving precision and by providing a deeper understanding of how to optimise a questionnaire and understand the results of a used questionnaire.^55^^,^^56^ Test-retest of the SA-SH-Ext could also be valuable, even if the SA-SH is stable over time.^38^ In a test-retest of the Danish version of the SA-SH there were promising results with a mean agreement percentage for the overall scale of 95.2% when allowing for a 1 point difference when measured at a 2 week interval.^51^

## FURTHER STUDIES RECOMMENDED IN THIS FIELD

Studies using the SA-SH-Ext in educational interventions for social educators and to assess social educator students´ perceived attitudes and readiness to address sexual health with future clients would show whether social educator students share similar response patterns to other health professional students. Assessing and understanding the students’ attitudes to addressing sexual health can be used to raise awareness among students of their knowledge gaps, but there is also a need to further research relevant educational strategies to address shown knowledge gaps. In addition, it would be of interest to adapt and psychometrically test SA-SH-Ext for social educators working in practice, both to enable comparisons between students and professionals and to follow the development within the profession over time.

## CONCLUSION

The psychometric assessment of the SA-SH-Ext show that the questionnaire is useful for measuring social educator students´ readiness to address sexual health in their future profession. The SA-SH-Ext can be used in research and for planning and measuring the results of educational interventions aiming to improve attitudes towards addressing sexual health in practice.

The psychometric assessment have revealed that the domains of the SA-SH-Ext should be revised according to the results of this study. The SA-SH-Ext is a valuable questionnaire for measuring the level of perceived readiness among social educator students in addressing sexual health, a field often neglected in health and care.

## AVAILABILITY OF DATA AND MATERIAL

Due to the nature of this research, participants of this study did not agree for their data to be shared publicly, so supporting data is not available in accordance with the ethical approval.

## AUTHOR CONTRIBUTIONS

GHL, LB, HG and KAJ all contributed to planning the project and writing the manuscript. GHL performed the data collection. KAJ carried out the statistical analysis.

## STATEMENT OF AUTHORSHIP

All authors contributed to planning the project and writing the manuscript. HL performed the data collection. KAJ carried out the statistical analysis.

## References

[1] Chrastina J, Večeřová H. (2020). Supporting sexuality in adults with intellectual disability—a short review. Sex Disabil.

[2] Dinwoodie R, Greenhill B, Cookson A. (2020). ‘Them two things are what collide together’: Understanding the sexual identity experiences of lesbian, gay, bisexual and trans people labelled with intellectual disability. J Appl Res Intellect Disabil.

[3] Dyer K, das Nair R. (2013). Why don't healthcare professionals talk about sex? A systematic review of recent qualitative studies conducted in the United kingdom. J Sex Med.

[4] Gilmore L, Chambers B. (2010). Intellectual disability and sexuality: Attitudes of disability support staff and leisure industry employees. J Intellect Dev Disabil.

[5] Lunde H. Hvordan møter høgskolen (HIOA) utfordringer knyttet til personer med utviklingshemming og seksualitet? 2013.

[6] Pelleboer-Gunnink HA, van Oorsouw WMWJ, van Weeghel J (2019). Stigma research in the field of intellectual disabilities:A scoping review on the perspective of care providers. Int J Dev Disabil.

[7] van den Bogaard KJHM, Nijman HLI, Embregts PJCM. (2019). Attributional styles of support staff working with people with intellectual disabilities exhibiting challenging behaviour. J Appl Res Intellect Disabil.

[8] Wilson NJ, Frawley P. (2016). Transition staff discuss sex education and support for young men and women with intellectual and developmental disability. J Intellect Dev Disabil.

[9] Leonardi-Warren K, Neff I, Mancuso M (2016). Sexual health: Exploring patient needs and healthcare provider comfort and knowledge. Clin J Oncol Nurs.

[10] Fennell R, Grant B. (2019). Discussing sexuality in health care: A systematic review. J Clin Nurs.

[11] McGrath M, Lynch E. (2014). Occupational therapists' perspectives on addressing sexual concerns of older adults in the context of rehabilitation. Disabil Rehabil.

[12] Haesler E, Bauer M, Fetherstonhaugh D. (2016). Sexuality, sexual health and older people: A systematic review of research on the knowledge and attitudes of health professionals. Nurse Educ Today.

[13] UNGeneralAssembly. Convention on the Rights of Persons with Disabilities: Resolution /adopted by the General Assembly, 24 January 2007. 2007.

[14] Barne-likestillings-oginkluderingsdepartementet. Konvensjon om rettighetene til mennesker med nedsatt funksjonsevne (Q-1199 B). Merkur-Trykk AS; 2013.

[15] Helse-ogomsorgsdepartementet. Snakk om det! Strategi for seksuell helse (2017-2022) (I-1175 B). Kord AS; 2016.

[16] Traeen B, Martinussen M. (2008). Attitudes toward sexuality among straight and queer university students from Cuba, Norway and South Africa. Scand J Psychol.

[17] Boey KW, Ng HN. (2020). Assessing sexual knowledge and sexual attitudes of nursing students: Implications for primary health care. Int J Public Health Res.

[18] Grace N, Greenhill B, Withers P. (2020). “They just said inappropriate contact.” What do service users hear when staff talk about sex and relationships?. J Appl Res Intellect Disabil.

[19] Kunnskapsdepartementet. Forskrift om nasjonal retningslinje for vernepleierutdanning. 2019.

[20] Grung RM. Vernepleieren: Fremtidsrettet og ettertraktet. In: Workers NUoSEaS, ed. Oslo: Norwegian Union of Social Educators and Social Workers; 2019.

[21] Lunde GH. (2013). Ansatte og temaet seksualitet: Hvilke utfordringer opplever ansatte i sitt arbeid når det gjelder voksne med intellektuell funksjonsnedsettelse og seksualitet?. Nordisk tidsskrift for helseforskning.

[22] Brask OD, Østby M, Ødegård A. (2016).

[23] Horndalen B, Torp TR (2006). Vernepleier – utdanning og yrke i et faglig og etisk perspektiv.

[24] Åker TH, Johnson MS. (2020). Sexual abuse and violence against people with intellectual disability and physical impairments: Characteristics of police-investigated cases in a Norwegian national sample. J Appl Res Intellect Disabil.

[25] Steans J, Duff S. (2020). Perceptions of sex offenders with intellectual disability: A comparison of forensic staff and the general public. J Appl Res Intellect Disabil.

[26] Murphy G, Withers P. (2020). Editorial for special issue on sexuality. J Appl Res Intellect Disabil.

[27] Sung S-C, Huang H-C, Lin M-H. (2015). Relationship between the knowledge, attitude, and self-efficacy on sexual health care for nursing students. J Prof Nurs.

[28] Gerbild H, Larsen CM, Junge T (2021). Danish health professional students’ attitudes towards sexual health – A cross-sectional survey. Sexual Medicine.

[29] Penwell-Waines L, Wilson CK, Macapagal KR (2014). Student perspectives on sexual health: Implications for interprofessional education. J Interprof Care.

[30] Papaharitou S, Nakopoulou E, Moraitou M (2008). Exploring sexual attitudes of students in health professions. J Sex Med.

[31] Areskoug-Josefsson K, Larsson A, Gard G (2016). Health care students’ attitudes towards working with sexual health in their professional roles: Survey of students at nursing, physiotherapy and occupational therapy programmes. Sex Disabil.

[32] Dağlı E, Uçtu AK, Reyhan FA (2020). Opinions of students in the field of health about the sexuality of individuals with disability. Sex Disabil.

[33] Gerbild H, Larsen C, Rolander B (2018). Does a two-week educational intervention change health care students’ attitudes towards addressing sexual health? – Results of a pilot study. Sex Disabil.

[34] Areskoug-Josefsson K, Rolander B. (2019). Understanding response patterns among healthcare professionals regarding their attitudes toward working with sexual health—Latent class analysis of the SA-SH. J Nurs Meas.

[35] Areskoug-Josefsson K, Rolander B. (2020). Value of performing a Rasch analysis on a reliable and valid instrument – case study of the SA-SH. J Nurs Meas.

[36] Felter CE. (2020). Assessing DPT students' self-perceived readiness to discuss sexual health before and after instruction from a patient-educator. J Phys Ther Educn.

[37] Gerbild H, Larsen CM, Rolander B (2016). Health care students’ attitudes towards addressing sexual health in their future professional work: Psychometrics of the danish version of the students’ attitudes towards addressing sexual health scale. Sex Disabil.

[38] Areskoug-Josefsson K, Juuso P, Gard G (2016). Health care students' attitudes toward addressing sexual health in their future profession: Validity and reliability of a questionnaire. Int J Sex Health.

[39] Areskoug-Josefsson K, Rolander B, Bülow P (2019). Swedish social work students’ attitudes toward addressing sexual health issues in their future profession. Sex Disabil.

[40] Areskoug-Josefsson K, Thidell F, Rolander B (2018). Prosthetic and orthotic students' attitudes toward addressing sexual health in their future profession. Prosthet Orthot Int.

[41] Areskoug-Josefsson K, Sjökvist M, Bülow PH (2019). Psychometrics of the students´ attitudes towards addressing sexual health scale for students in social work. Social Work Education.

[42] Lunde GH, Bakke A, Areskoug-Josefsson K. (2020). Piloting a research-oriented teaching model in a bachelor program for social educators – A way to increase competence in research methodology and sexual health?. Uniped.

[43] DeVon HA, Block ME, Moyle-Wright P (2007). A psychometric toolbox for testing validity and reliability. J Nurs Scholarsh.

[44] Mokkink LB, Terwee CB, Patrick DL (2010). The COSMIN study reached international consensus on taxonomy, terminology, and definitions of measurement properties for health-related patient-reported outcomes. J Clin Epidemiol.

[45] Mokkink LB, de Vet HCW, Prinsen CAC (2018). COSMIN risk of bias checklist for systematic reviews of patient-reported outcome measures. Qual Life Res.

[46] Terwee CB, Bot SD, de Boer MR (2007). Quality criteria were proposed for measurement properties of health status questionnaires. J Clin Epidemiol.

[47] Hair J, Anderson R, Tatham R, Black W (1998). Multivaliate Data Analysis.

[48] Shakespeare T, Richardson S. (2018). The sexual politics of disability, twenty years on. Scand J Disabil Res.

[49] Royal K. (2016). "Face validity" is not a legitimate type of validity evidence!. Am J Surg.

[50] Bolarinwa OA. (2015). Principles and methods of validity and reliability testing of questionnaires used in social and health science researches. Niger Postgrad Med J.

[51] Prebble KS, Gerbild H, Abrahamsen C. (2021). Content validity and reliability of the danish version of health care students’ attitudes towards addressing sexual health: A psychometric study. Scand J Caring Sci.

[52] Brooks H, Llewellyn CD, Nadarzynski T (2018). Sexual orientation disclosure in health care: A systematic review. Br J Gen Pract.

[53] Grung RM. (2016). The role of the Norwegian social educator. Learn Disabil Pract.

[54] Morton SM, Bandara DK, Robinson EM (2012). In the 21st century, what is an acceptable response rate?. Aust N Z J Public Health.

[55] Bond TG, Fox CM (2015).

[56] Boone WJ. (2016). Rasch analysis for instrument development: Why, when, and how?. CBE Life Sciences Education.

